# Reviewing evidence on complex social interventions: appraising implementation in systematic reviews of the health effects of organisational-level workplace interventions

**DOI:** 10.1136/jech.2007.071233

**Published:** 2008-08-21

**Authors:** M Egan, C Bambra, M Petticrew, M Whitehead

**Affiliations:** 1 Medical Research Council Social and Public Health Sciences Unit, University of Glasgow, UK; 2 Department of Geography, Wolfson Research Institute, Durham University, UK; 3 Public and Environmental Health Research Unit, London School of Hygiene and Tropical Medicine, UK; 4 Division of Public Health, University of Liverpool, UK

## Abstract

**Background:**

The reporting of intervention implementation in studies included in systematic reviews of organisational-level workplace interventions was appraised. Implementation is taken to include such factors as intervention setting, resources, planning, collaborations, delivery and macro-level socioeconomic contexts. Understanding how implementation affects intervention outcomes may help prevent erroneous conclusions and misleading assumptions about generalisability, but implementation must be adequately reported if it is to be taken into account.

**Methods:**

Data on implementation were obtained from four systematic reviews of complex interventions in workplace settings. Implementation was appraised using a specially developed checklist and by means of an unstructured reading of the text.

**Results:**

103 studies were identified and appraised, evaluating four types of organisational-level workplace intervention (employee participation, changing job tasks, shift changes and compressed working weeks). Many studies referred to implementation, but reporting was generally poor and anecdotal in form. This poor quality of reporting did not vary greatly by type or date of publication. A minority of studies described how implementation may have influenced outcomes. These descriptions were more usefully explored through an unstructured reading of the text, rather than by means of the checklist.

**Conclusions:**

Evaluations of complex interventions should include more detailed reporting of implementation and consider how to measure quality of implementation. The checklist helped us explore the poor reporting of implementation in a more systematic fashion. In terms of interpreting study findings and their transferability, however, the more qualitative appraisals appeared to offer greater potential for exploring how implementation may influence the findings of specific evaluations. Implementation appraisal techniques for systematic reviews of complex interventions require further development and testing.

The case has been made for providing policy-makers with synthesised, detailed and robust accounts of the implementation of effective interventions in order to make better progress in tackling population morbidities and inequalities.^1^ Advocates of a staged approach to the development and evaluation of complex interventions have also stressed the importance of accurately defining interventions and promoting effective implementation.^2^ Implementation refers to the design and delivery of interventions.^3^^–^6 The way an intervention is implemented may influence its outcomes, and evaluations that do not take this into account risk (for example) misinterpreting negative outcomes that result from poor implementation as evidence that interventions are inherently ineffective.^7^ ^8^ We developed a tool to appraise the quality of reporting of implementation and applied this tool to four systematic reviews of complex intervention evaluations affecting the workplace.

## Implementation and complex interventions

Researchers and policy-makers have called for evidence from systematic reviews of social interventions affecting so-called “upstream” health determinants such as employment, housing, transport, etc.^9^ ^10^ Such interventions are often complex and difficult to evaluate.^11^ ^12^ They may involve multiple, context-specific interventions and an unstandardised approach to implementation.^13^

In our experience of conducting systematic reviews of “upstream” interventions, it is often difficult from the reporting of a complex intervention evaluation to determine: (1) what exactly the intervention entailed; (2) whether the intervention was implemented fully or adhered to good practice guidelines; and (3) whether there were confounding factors in the wider social context that would affect the outcome of the intervention.^14^^–^21 This contrasts with reports of less complex interventions in which (1) the intervention is clear (eg a specific drug); (2) intervention delivery was prescribed through a detailed protocol; and (3) at least some attempt was made from the planning stage onwards to identify and reduce bias associated with key confounders.

## Implementation appraisal

Implementation appraisal is not a new concern.^22^^–^29 Some systematic reviews have considered whether interventions were delivered as prescribed by the study protocol (“treatment integrity” or “programme adherence”).^30^ However, appraisal tools used by systematic reviewers usually focus on the methodological characteristics of primary studies rather than implementation issues.^30^ ^31^ Such tools often take the form of checklists, although the practice of using checklist scores to appraise studies is problematic, leading some to advocate alternative approaches.^31^ ^32^

Systematic reviews that attempt to rigorously appraise the implementation of complex interventions are the exception rather than the rule. A recent review of community-based injury prevention initiatives, which included appraisals of evidence on implementation, found that reporting of implementation was poor.^33^

We developed and incorporated an appraisal checklist into four systematic reviews of organisational-level workplace interventions, along with a less structured exploration of textual accounts of implementation in the included studies.^17^^–^20 The checklist covered reporting of intervention design (including whether or not interventions were specifically designed to affect employee health), target population, delivery, psychosocial factors and the characteristics of population subgroups differentially affected by the interventions. Our primary aim was to appraise the reporting of implementation in primary studies; our study also considered whether or not there was evidence to suggest that higher standards of reporting were an indication of greater methodological rigour.^34^

## METHODS

The four reviews that incorporated our appraisal tool synthesised evidence on the health effects of (1) workplace interventions to increase employee control and participation in decision-making;^17^ (2) changes to team structures and work allocation affecting employees’ day-to-day tasks;^18^ (3) the health effects of instigating compressed working weeks;^19^ and (4) shift work interventions.^20^ Table 1 summarises the intervention types in these reviews. Their methods and outcomes have been described elsewhere.^17^^–^20

**Table 1:** Details of the interventions included in the four systematic reviews

Interventions	Descriptions
Employee participation review^15^	
Employee participation committees	Employee representative committees (described variously, eg action teams, problem solving committees, etc). These often focus on identifying and suggesting ways of overcoming workplace stressors. Some committees are led by external facilitators and/or have managerial representation.
Flexible working hours	Employees are given more control over choosing their working hours.
Participation and individual-level interventions	Employee committees combined with individual-level health promotion, education and behaviour programmes: such as anti-smoking or physical activity interventions and training in relaxation techniques, stress reduction and communication skills.
Participation and ergonomic interventions	Participatory committees combined with ergonomic interventions, ie attempts to reduce physical discomfort and workplace injuries by modifying physical environments (including technological improvements) and advising on posture and lifting.
Task restructuring review^16^	
Production line	Production line interventions that increase the variety of tasks performed by a worker, increase the skills utilised and place more responsibility on individual workers.
Primary nursing	Increasing the skills utilised by workers by increasing the variety of work tasks. Primary nursing and personal caregiving are patient-orientated care systems in which each patient is assigned to an individual nurse/carer; the nurse/carer takes 24-hour responsibility for the care of that patient including the planning and quality of the care provided.
Team working	Workers are given more collective responsibility and decision-making power within the team, but responsibility is not shared and supervisory structures remain in place.
Lean production	Employee workloads are maximised, wasted time is reduced, tasks are distributed within the team, and work standards are determined by the employees themselves rather than solely by management.
“Just in time”	“Just in time” requires that products are made “just in time” to be sold—no stockpiling of products. Work groups, not individuals, are given autonomy and responsibility for specific tasks in order to achieve the required production flow.
Autonomous work groups	Autonomous work groups are characterised by employee self-determination and involvement in the management of day-to-day work (including control over pace, task distribution and training and recruitment).
Compressed working week review^17^	
Compressed working week (CWW)	Hours worked per day are increased while the days worked are decreased in order to work the standard number of weekly hours in less than 5 days, eg the *12-hour CWW* involves four 12-hour shifts (day, night) over 4 days with 3 or 4 days off. Under a *10-hour CWW*, four 10-hour shifts are worked followed by 3 days off. The *Ottawa* system consists of three or four 10-hour morning or afternoon shifts for 4 days and 2 days off followed by a block of seven 8-hour nights and 6 days off.
Shift work review^18^	
Changes affecting shift rotation	eg 1. Changing from slow to fast rotation: a change from six or seven consecutive shifts of the same type to a maximum of three or four.
	eg 2. Changing from backward (night, afternoon, morning) to forward (morning, afternoon, night) rotation or vice versa.
	eg 3. Changing from a rotating shift system to a permanent shift system.
Changes affecting night work	eg 1. Removal of night shifts.
	eg 2. Increasing the rest period before the rotation onto night shift.
	eg 3. Reduction in the number of consecutive night shifts.
Later start and finish times	Starting and finishing shifts 1 hour (in the studies we identified) later.
Weekend shift changes	Continuous (weekends on) to discontinuous shift system (weekends off) or vice versa.
Decreased hours	Decrease in shift length (eg from 8 hours to 6 hours).
Self-scheduling	Self-scheduling enables individual shift workers to have some control over which shifts they work, their start times or when their rest days occur.
Combined shift interventions	Combinations of the above, most usually shift rotation combinations, eg change to fast and forward rotation.

Our original checklist contained 28 criteria. These criteria were adapted from a number of sources, particularly Rychetnik *et al*, whose work had prompted our initial interest in implementation.^3^ ^4^ ^27^ ^35^^–^38 Two reviewers (ME and CB) piloted this checklist independently using 12 studies (taken from the participation and task restructuring reviews). On comparing their pilot appraisals, the reviewers agreed that the checklist had been difficult to interpret and apply consistently, and they criticised both its content and its face validity. The reviewers ascribed these problems to the checklist being unclear (often because criteria had been adapted from other contexts). The pilot checklist also coped poorly with ambiguities in reports of implementation (often, the answers to specific checklist criteria were implied rather than explicitly stated in the brief reports of implementation we identified—and it was often difficult to agree on the point at which reviewers could distinguish mere implication from reported fact). We decided that it would be preferable to work with a smaller number of broader criteria, and hence we shortened the checklist.

The final checklist included 10 criteria (response: yes/no). Studies were categorised by an implementation appraisal score (out of 10—one point for the presence of each criterion), distinguishing the “lowest”, “intermediate” and “higher” scoring studies. The checklist is presented in table 2.

**Table 2:** Thematic checklist for the appraisal of the reporting, planning and implementation of workplace interventions

Theme	Checklist question for workplace reviews
1. Motivation	Does the study describe why the management decided to subject the employee population to the organisational change?
2. Theory of change	Was the intervention design influenced by a theory of change describing the proposed pathway from implementation to health outcome?
3. Implementation context	Does the study provide any useful contextual information relevant to the implementation of the intervention (eg political, economic or managerial factors)?
4. Experience	Does the study establish whether those implementing the intervention had appropriate experience (eg had the implementers conducted similar interventions before; or, if managers/employees were involved, were they appropriately trained for the new roles)?
5. Planning consultations	Is there a report of consultation/collaboration processes between managers, employees and any other relevant parties during the planning stage?
6. Delivery collaborations	Is there a report of consultation/collaboration processes between managers, employees and any other relevant parties during the delivery stage?
7. Manager support	Were on-site managers/supervisors supportive of the intervention (eg do the authors comment on managers’ views of intervention)?
8. Employee support	Were employees supportive of the intervention (eg do the authors comment on employees’ views of intervention)?
9. Resources	Does the study give information about the resources required in implementing the intervention (eg time, money, people, equipment)?
10. Differential effects and population characteristics*	Does the study provide information on the characteristics of the people for whom the intervention was beneficial, and the characteristics of those for whom it was harmful or ineffective?

*Note that, while consideration of differential effects involves analysis of outcomes, it also provides contextual information on the characteristics of population subgroups. This is of interest when exploring the transferability of research findings and the mechanisms by which some interventions affect different types of people in different ways. Hence, we regard this issue as relevant to explorations of implementation and context (as well as to outcome analysis).

Two reviewers (ME and CB, or CB and MP) independently applied the checklist to all the studies included in the four systematic reviews. Differences were resolved through consultation. We then used cross-tabulations to explore relationships between quality appraisal scores from our checklist and data on evaluation study designs, and with psychosocial and health outcomes (previous studies have suggested that more rigorous evaluations may be less likely to report positive outcomes).^34^ We also explored whether reporting of implementation differed by date of publication (ie whether or not reporting has improved in recent years) and type of publication (ie whether reporting is better or worse in peer-reviewed journals compared with other forms of publication).

Reported text that described implementation processes were also extracted from each study by one reviewer and checked by another to aid a less structured analysis of reporting of implementation for each review. We considered relevant data first on a case-by-case basis and explored the interactions between reported planning and implementation characteristics, contexts and outcomes. We discussed patterns and idiosyncrasies across different studies and synthesised key findings using a narrative approach. From this less structured process, we gained some insights into how a minority of authors explained outcomes in terms of implementation characteristics.

## RESULTS

Implementation appraisals were conducted on a total of 103 studies (references can be obtained from the original reviews).^17^^–^20 Twenty-one studies were identified in the task restructuring review, 18 studies in the employee participation review, 40 studies in the compressed working week review and 26 studies in the shift work review.^17^^–^20 Two studies appeared in two reviews. In table 3, the numerical implementation scores are summarised for all studies and, in table 4, examples of summaries of implementation appraisals are presented for the higher scoring studies from each of the four reviews.

**Table 3:** Numerical summary of the results of the implementation appraisal checklist

	All reviews (excluding duplicates)	Task variety	Employment participation	Compressed working week	Shift work
Total number of studies	103	21	18	40	26
Mean implementation score	2.6	2.6	3.4	2.3	2.4
Median implementation score	3	3	4	2	2.5
Studies with lowest implementation score (0,1,2)	51	9	4	26	13
Studies with intermediate implementation score (3,4)	38	10	10	8	11
Studies with higher implementation score (⩾5)	14	2	4	6	2

**Table 4:** Examples of implementation appraisal summaries (higher scoring studies only)

Citation	Intervention, study design and population details	Implementation details*	Score
Employee participation review			
Park *et al* (2004)^39^	Participatory committee to improve team communication and cohesiveness, work scheduling, conflict resolution and employee rewards	(1) Researcher initiated to act as a buffer against the adverse effects of recession and uncertainty	6
	Prospective repeat cross-sectional study	(2) Explicitly inspired by theories of psychosocial work reorganisation	
	All employees, retail store, USA	(3) Implementation took place during a period of recession and uncertainty	
		(5) Professional facilitator assisted with delivery	
		(6) Employee representative liaised with management and employees	
		(10) Psychosocial improvements for black and Hispanic, but not white, employees	
Mikkelsen and Saksvik (1999)^40^	Conference on working conditions followed by supervisor and employee work groups meeting 2 hours a week, nine times: intervention was moderated by consultants	(1) To improve workplace health	6
	Prospective cohort study with comparison group	(2) Explicitly inspired by theories of psychosocial work reorganisation	
	Manual and clerical workers, Post Office depot, Norway	(3) Company undergoing downsizing for financial reasons	
		(5) Researchers, managers and union representatives helped design the intervention	
		(7) Management supported the intervention	
		(8) Union representatives supported the intervention, but the authors report that, in one department, the intervention was neither successfully implemented nor effective, because steering group members lost interest, and personnel were relocated or made redundant	
Task restructuring review			
Wall *et al* (1990)^41^	Increased operator control on production line	(1) Introduced to increase staff performance	5
	Prospective cohort	(2) Explicitly inspired by theories of psychosocial work reorganisation	
	Manual workers, factory floor, UK	(4) Training was provided	
		(6) Representative of employees of all grades, and the researchers were involved in a working party overseeing the implementation of the intervention	
		(8) Some employees were resistant to the intervention	
Wall *et al* (1986)^42^	Autonomous work groups	(1) Intervention occurred in a purpose-built factory that was designed with increasing factory floor responsibility and job redesign in mind	5
	Prospective cohort	(2) Underpinned by theory about job redesign	
	Manual and shop floor supervisors, factory floor, UK	(4) Training on intervention was provided	
		(6) Researchers were not involved in the design or implementation of the intervention	
		(8) Employee support for the intervention was mixed	
Compressed working week review			
Williams (1992)^43^	Six/seven 8-hour shifts, 2/4 days off to three/four 12-hour shifts, 2–7 days off	(1) Intervention initiated by staff to improve their work/life balance. 90% of staff were dissatisfied with the old system and other local factories had started using 12-hour shifts	7
	Prospective cohort	(3) Pressure from staff led to a management review of different shift schedules with the most popular schedule adopted. 83% voted for the implemented system	
	Operators, chemical plant, USA	(4) Managers went on “fact finding” visits to 12-hour factories to learn about safety implications and how best to implement the change	
		(5) Staff input central to the planning and consultation process	
		(6) Key delivery collaborations between staff, union and managers aided implementation	
		(7) Managers were supportive of the intervention	
		(8) Union was supportive of the intervention	
Wootten (2000a,b)^44^,^45^	7.5-hour to 12-hour shifts	(1) Introduced to improve staff health and well-being	7
	Retrospective cohort	(2) Explicitly inspired by theories of psychosocial work reorganisation	
	Nurses, hospital, UK	(4) Colleagues who had implemented similar changes elsewhere were consulted	
		(5) Staff were consulted over the change	
		(6) Implemented via collaborations between staff, supervisors and unions	
		(7) Managers were initially hesitant but then agreed	
		(8) 75% of staff agreed to a pilot	
Brinton (1983)^46^	Five 8-hour shifts, 2 days off to four 12-hour shifts, 3/4 days off	(1) Workers’ idea	7
	Retrospective repeat cross-section	(2) Explicitly inspired by theories of psychosocial work reorganisation	
	Wood yard workers, paper mill, USA	(3) Flexibility needed by both union and management to get the new system implemented	
		(5) New system designed and agreed with the union	
		(6) Committee set up between the union and managers to monitor safety in the new system	
		(7) Supported by supervisors. Company management agreed that they would implement the change if a majority of the workforce supported it	
		(8) Supported by the union	
Changes to shift work			
Gauderer and Knauth (2004)^47^	Self-scheduling of shifts	(1) Introduced to improve the ergonomic design of shifts by involving drivers in their own scheduling	5
	Prospective cohort with comparison group	(2) Explicitly inspired by theories of psychosocial work reorganisation	
	Bus drivers, public transport depot, Germany	(4) Those involved in implementation collected information from other companies that had experienced a similar intervention. Workers attended training workshops to learn how to design their own schedules	
		(5) Staff, managers and researchers involved in designing the system	
		(8) Workers’ council voted in favour of the change and, at the end of the 1-year trial period, workers voted to keep the new system	
Kandolin and Huida (1996)^48^	Slow to fast rotation; backward to forward rotation; self-scheduling of shifts	(1) Introduced to reduce fatigue by decreasing the number of “quick returns” and changing to a forward rotation. Aimed to increase the role of midwives in their own scheduling	6
	Prospective cohort with comparison group	(3) Only a third of the midwives said that they had actually experienced a change to forward rotation, but more experienced less quick returns on the new system. A higher proportion of staff now participated in their own scheduling	
	Midwives, hospital, Finland	(4) Managers had previous experience	
		(5) Managers carried out the rescheduling	
		(6) Managers carried out the rescheduling	
		(8) 55% said they preferred the old system because ofthe longer continuous free time	

*1 = Motivation; 2 = Theory; 3 = Context; 4 = Experience; 5 = Planning; 6 = Delivery; 7 = Managerial response; 8 = Employee response; 9 = Resources; 10 = Differential effects and population characteristics (see table 2 for more details).

### Summary of implementation appraisals

Most studies achieved low scores (see table 3). The median score was 3 out of 10 (range = 0 to 7; lower and upper quartiles  = 1 and 4). This varied slightly between reviews (from 2 to 4). The median score was 3 for studies published between 1996 and 2000 and 2 for studies published between 2001 and 2006, and between 1991 and 1995 and before 1991.

As few studies achieved a high implementation score, we have categorised the studies as follows: 14 “higher” scoring studies (scoring ⩾5 in our implementation appraisal), 38 “intermediate” scoring studies (scoring 3 or 4 in our appraisal) and 51 “lowest” scoring studies (scoring <3).

The most commonly reported implementation themes were “motivation for intervention” (table 2, criteria 1—appearing in 76% of included studies) and employee support of the intervention (criteria 8—appearing in 54% of the studies). All the other themes were reported in less than a third of the total studies. Criteria 10 (differential effects/population characteristics) was only reported in 8% of the studies, while no study described resourcing, costs or cost–benefits of interventions (criteria 9).

### Type of publication

Forty-nine included studies were published in peer review health journals, 41 in other peer review journals (mainly social science, occupational and managerial studies journals) and 13 in edited books or theses. Twelve per cent of articles from health journals received higher implementation scores compared with 15% of studies from both other journals and books or theses. Forty-seven per cent of articles from health journals received lower implementation scores compared with 51% of studies from other journals and 54% from books or theses.

### Implementation and study design

Implementation appraisal scores were not useful predictors of robust study designs. We identified 32 prospective cohort studies with appropriate controls and have classed these as the most robust study designs: 36% of the studies with “higher” implementation scores were “most robust” compared with 45% of studies with intermediate scores and 20% of studies with low scores.

### Implementation and health effects

All 103 studies included in the reviews evaluated at least one health outcome.^17^^–^20 We have categorised the studies as follows: (1) those that reported at least one positive health outcome and no negative outcomes (n = 47); (2) those that reported at least one negative health outcome and no positive outcomes (n = 14); and (3) those that report conflicting health outcomes (positive and negative) or reported little/no change in all the health outcomes measured (n = 42).

We found no conclusive evidence that better reporting of implementation might be associated with positive health outcomes. There was a similar range of implementation scores for both the 47 studies with positive outcomes (47% scored <3, 40% scored 3 or 4, and 13% scored ⩾5) and the 42 studies with conflicting/little change in outcomes (45% scored <3, 40% scored 3 or 4, and 14% scored ⩾5). Fourteen studies reported negative outcomes, of which 84% scored <3, 15% scored 3 or 4, and none scored ⩾5 on the implementation checklists.

### Unstructured appraisals of implementation

We extracted textual data on implementation from all the included studies for less structured, more qualitative appraisals. However, we focus on the 14 studies with negative health outcomes.

Implementation reporting tended to be brief and anecdotal. It was often unclear how authors had obtained their information about implementation and whether they had taken steps to avoid bias or error. These (important) objections aside, our more qualitative approach to implementation appraisal did appear to uncover potential explanations for how the implementation characteristics of some studies may have contributed to negative outcomes.

In the participation review, we found that the only two studies with negative health outcomes evaluated participatory interventions that had been implemented in workplaces undergoing organisational downsizing.^17^ We found that in the “task variety” review, negative health outcomes were more likely to result from interventions that were motivated for business reasons (managerial efficiency, productivity, cost, etc) rather than by employee health concerns.^18^ However, the studies identified for the compressed working week and the shift work reviews provide evidence of positive, negative or “little change” outcomes resulting from interventions regardless of whether they were motivated by business concerns, health concerns or pressure from employees.^19^ ^20^

## DISCUSSION

Promoting effective implementation is regarded as a key stage in the design and evaluation of complex interventions, and syntheses of evidence from such evaluations should incorporate data on implementation.^1^ ^2^ We incorporated implementation data into four systematic reviews of workplace interventions, using both a specially developed checklist for measuring reporting of intervention design and implementation and a more qualitative approach to assessing such reports. We found that reporting of implementation was generally poor. Our experience led us to reflect upon whether a checklist is the best tool for appraising implementation, particularly as our qualitative approach was easier to conduct and, we conclude, more useful than the checklist-based approach.

### Quality of reporting

In most cases, authors of included studies presented brief and anecdotal reports of implementation. We identified few descriptions of how authors obtained information about implementation, whether any prior code of good practice existed against which the quality of implementation could be measured, and whether any attempts were made to prevent biased reporting of implementation. Roen and colleagues recently published details of their attempts to appraise the implementation of injury prevention interventions, which identified similarly poor standards of reporting.^33^ However, they found that studies with methodologically stronger designs tended to provide poorer descriptions of implementation. We found no clear evidence of this relationship in our reviews.

Our checklist-based appraisals did find that most included studies provided some information about what motivated the implementers to deliver the intervention, and whether employees supported them. However, data on cost-effectiveness and differential effects on population subgroups were rarely reported, despite the widely stated view that research to inform public health policy and practice should provide evidence on these issues.^11^ ^12^ We also found that reporting of implementation varied little by year or type of publication.

We also took a less structured (and less score-focused) approach to identifying reported data on implementation appraisal. This did identify some potential explanations for how implementation may have affected psychosocial and health outcomes, eg organisational downsizing, lack of management support and the aim of increasing individual productivity without regard to employee well-being were all offered as explanations for negative results. These issues were usually described anecdotally within the studies, yet they often provided the most plausible explanations for negative outcomes available to reviewers.

We note that other systematic reviewers have employed more qualitative approaches to implementation appraisal.^29^ Our own experience now leads us to advocate variations on this approach, perhaps as an adjunct to the use of implementation checklists.

### Limitations

More methodological work is required to develop our approach (and alternative approaches)^33^ to implementation appraisal: in particular to test inter-rater reliability and validity (the lack of such tests is a limitation to this study). We would focus our efforts on developing and testing qualitative implementation appraisal methods as we believe these may potentially provide greater insights than a checklist-based approach.

We do not rule out the possibility that a systematic review checklist could be developed to assist with implementation appraisals but, in our experience, this approach was problematic. The checklist we developed assessed reporting of implementation—this is not the same as appraising the quality of implementation, but good reporting is one prerequisite for such an appraisal.^1^ Our checklist therefore helped to demonstrate the urgent need for improved reporting, but did not help us to understand how implementation affected outcomes.

It should also be remembered that this paper only examines reviews of employment interventions. The generalisability of these findings depends on the degree to which employment researchers tend to report implementation differently from or similarly to researchers working in other fields.

We also note that our implementation checklist analysis explored psychosocial and health outcomes. While it is legitimate for public health researchers to be particularly interested in outcomes relevant to their field, we recognise that complex interventions such as those included in our reviews often have other outcomes (eg financial, managerial) of equal or greater importance to the implementers than health outcomes.

### Quality of implementation

As stated above, our checklist was not designed to appraise quality of implementation. Even if we could have directly appraised the quality of implementation, the checklist scores would still have been problematic. Summary appraisal scores reveal the number and variety, but not the importance, of reported implementation characteristics. It may only take one flaw in the implementation to cause an intervention to fail, so a high intervention score is no guarantee of effectiveness.^32^

Furthermore, the development of a detailed checklist for measuring quality of implementation requires an *a priori* knowledge of the criteria that will distinguish well-implemented interventions from poorly implemented interventions.^1^ This may be feasible in some areas of research, when there is a strong consensus regarding standards of best practice, but that consensus does not always exist for every type of intervention. We attempted to develop such a list but quickly realised that the included interventions were too varied and there was often no clear way of prescribing in detail what constituted good or bad practice. We suspect that this uncertainty over best practice may increase with the complexity of an intervention, particularly if the intervention is flexible in design and context specific.

For example, what resources are sufficient to adequately resource an intervention? Is collaboration with employees always desirable, or can interventions achieve similar or better results if they are imposed by managers taking a “strong leader” approach? It may be desirable for people managing implementation processes to have appropriate experience, but “appropriate” needs to be defined: must managers have prior experience of delivering specific interventions, or is their general role in management to be regarded as appropriate enough?

The answers to all these questions seem to us to depend on the intervention and specific circumstances.

**What is already known on this subject**

- Systematic reviews have been advocated as a means of identifying and appraising evidence on the health effects of complex social interventions.
- Implementation should be an important feature of these types of systematic review.
- However, to date, reviewers have often placed more emphasis on appraising the methodological characteristics of evaluations rather than the intervention itself and how it is implemented.
- Implementation appraisal tools have therefore remained relatively underdeveloped in the systematic review literature, especially as regards more complex social interventions.

**What this study adds**

- Aside from highlighting the lack of reporting of implementation issues in primary studies, this study also reveals which aspects of implementation were most commonly reported.
- We do not recommend our checklist as a means of appraising how implementation influences the outcomes of interventions. Implementation appraisal may be best achieved through less structured and more qualitative approaches.
- Future evaluations of implementation need to incorporate more contextual, qualitative information.

**Policy implications**

Primary studies and systematic reviews that are intended to influence policy and practice risk making erroneous recommendations if the quality of intervention implementation is not more robustly appraised.

### Conclusion

Guidance on improving the reporting of implementation has been published elsewhere along with the recommendation that “adding simple criteria to reporting standards will significantly improve the quality and usefulness of published evidence and increase its impact on public health program planning”.^1^ Such guidance may need to be adapted to suit specific interventions, and our own checklist includes criteria that may be useful when reporting workplace interventions. However, we would advise caution against assuming that appraising the implementation of complex interventions is a simple matter. The appraisal tool we developed—like other appraisal tools—could only assess how well the implementation process was reported, rather than the quality of the process itself and, in most cases, reporting was poor. We also lacked detailed criteria on what constitute well-implemented workplace interventions that could safeguard or improve employee health.

Nonetheless, information on implementation and context is crucial for a nuanced assessment of the impact of complex interventions. Improvements in the reporting and appraisal of such information are overdue.
